# Integrated and independent evolution of heteromorphic sperm types

**DOI:** 10.1098/rspb.2013.1647

**Published:** 2013-10-22

**Authors:** Allen J. Moore, Leonardo D. Bacigalupe, Rhonda R. Snook

**Affiliations:** 1 Department of Genetics, University of Georgia, Athens, GA 30602, USA; 2 Centre for Ecology and Conservation, College of Life and Environmental Sciences, University of Exeter, Cornwall Campus, Penryn TR10 9EZ, UK; 3 Instituto de Ciencias Ambientales y Evolutiva, Facultad de Ciencias, Universidad Austral de Chile, Casilla 567, Valdivia, Chile; 4 Department of Animal and Plant Sciences, University of Sheffield, Sheffield S10 2TN, UK

**Keywords:** *Drosophila pseudoobscura*, functional integration, quantitative genetics, modularity, evolvability

## Abstract

Sperm are a simple cell type with few components, yet they exhibit tremendous between-species morphological variation in those components thought to reflect selection in different fertilization environments. However, within a species, sperm components are expected to be selected to be functionally integrated for optimal fertilization of eggs. Here, we take advantage of within-species variation in sperm form and function to test whether sperm components are functionally and genetically integrated both within and between sperm morphologies using a quantitative genetics approach. *Drosophila pseudoobscura* males produce two sperm types with different functions but which positively interact together in the same fertilization environment; the long eusperm fertilizes eggs and the short parasperm appear to protect eusperm from a hostile female reproductive tract. Our analysis found that all sperm traits were heritable, but short sperm components exhibited evolvabilities 10 times that of long sperm components. Genetic correlations indicated functional integration within, but not between, sperm morphs. These results suggest that sperm, despite sharing a common developmental process, can become developmentally and functionally non-integrated, evolving into separate modules with the potential for rapid and independent responses to selection.

## 1. Introduction

Sperm are the most diverse cell type known, their morphology rapidly evolving among populations and species [1]. Because a sperm's proximate function is clear—fertilization—aspects of sperm morphology are predicted to have diverged as a consequence of natural selection on this function [1,2]. However, from an evolutionary perspective, the causes and consequences of the extraordinary sperm diversification are still the subject of much debate and study. The current evolutionary focus is on the role of postcopulatory sexual selection in generating diversity of sperm morphology [3–7]. In most studies, the form and extent of genetic variation and covariation that might shape sperm evolution is relatively unknown and little studied [8] despite it being necessary to fully understand rapid sperm evolution.

There are reasons to suspect that genetic variation and covariation of sperm components might be constrained, thus impacting evolution. The different sperm morphological structures (i.e. acrosome and nucleus (head), flagellum) are expected to work together to ensure fertilization in the distinctive postcopulatory environment of the species studied. That is, separate sperm components within-species should have a joint impact on fertilization performance, and thus be functionally integrated, as are other morphological units that act in common to influence a common component of fitness [9]. Thus, at the individual sperm level, selection on sperm morphological components may act to maintain functional integration of component parts of sperm for fertilization. Such functional integration is expected to be reflected in developmental integration at the individual level and genetic integration at the population level, given that the sperm components need to be inherited together [9,10]. Genetic integration is indicated by significant genetic covariances between sperm components. Strong stabilizing selection owing to the requirements for successful fertilization is further expected to be reflected in low levels of genetic variation and therefore low evolvability, *e_*μ*_*, which measures the potential for a response to directional selection [11,12].

The hypothesis of morphological and functional integration of sperm traits presents a problem for the expected rapid sperm morphological diversification by postcopulatory sexual selection. The responsiveness of sperm to selection, reflected by the species-specific diversity and responses to artificial selection [13,14], suggests that sperm components should exhibit sufficient additive genetic variation and evolvability. The conundrum, then, is twofold: how can sperm be so extraordinarily diverse when functional constraints may be operating, and how can individual sperm components evolve so rapidly?

One hypothesized mechanism to counteract constraints arising from functional integration is to reduce genetic or developmental linkages between modules. The concept of modularity describes developmental patterns and is also used to help understand evolutionary processes [15,16], because traits in separate modules can evolve independently. In a multivariate quantitative genetics context, modularity and thus the potential for independent evolution, is indicated whether the genetic covariance structure shows significant covariation within the modules but little statistical association between modules [16].

One of the most extreme examples of variation in sperm morphology is that of sperm heteromorphism, a taxonomically widespread phenomenon in which males produce two (or more) different types of sperm in the same testes [2]. These two sperm types are transferred in a single ejaculate, and although the functional significance of each sperm type is not always clear [2], it is expected that together they enhance the likelihood of fertilization [17]. For example, in silkworm moths, *Bombyx mori*, the presence of both the fertilizing (eusperm) type and the non-fertilizing (parasperm) type appear to be required for full fertility [18]. In *Drosophila pseudoobscura*, short parasperm appear to protect long eusperm viability in the spermicidal female reproductive tract [19,20]. Thus, in these systems, the performance of eusperm is potentially dependent on the presence of parasperm. Such a relationship extends the common definition of functional integration beyond the typical morphological one [21]. Given that both sperm types appear to be required for maximum fertility in heteromorphic species, then following the logic of functional integration, sperm should be genetically integrated both within, and between, types.

While each sperm type in *D. pseudoobscura* is produced and develops simultaneously within the same testes, each sperm morph develops separately in sperm bundles (each bundle producing 128 sperm of only one type) derived from a primary spermatocyte [22,23]. Thus, the two sperm types share a developmental environment within the testes but develop separately within that environment. This suggests that the two sperm types may be different modules, capable of independent evolution, and therefore patterns of genetic and developmental integration would occur within, but not between, each sperm type.

Here, we tested for correlated evolution of the two sperm types to determine whether (or not) sperm types are functionally integrated and examined the potential for sperm to evolve. We examined the pattern of genetic covariation to test whether the sperm types were genetically integrated, as predicted whether there is repeated co-selection of functionally integrated traits. If there is functional integration across sperm types, then there should be non-zero genetic correlations between eusperm and parasperm. These correlations should be especially strong if there is developmental integration [9,10]; for example, different sperm types derive from the same progenitor cells. We also calculated evolvabilities [11,12] to examine the potential for directional evolutionary change in sperm components.

## 2. Methods

### (a) Breeding design

We used a standard paternal half-sibling breeding design to quantify the genetic (co)variances of sperm traits in our laboratory population of *D. pseudoobscura*. Further details on this can be found in Snook *et al*. [24]. The population we analyse has been used for experimental evolution studies in which sexual selection has been manipulated. While previous works have found no phenotypic response in sperm traits [24,25], we still include line (*n* = 3) as a fixed effect in a mixed model design in all of our analyses.

For each line, virgin flies used as parents in the breeding design were collected by CO_2_ anaesthesia and housed in single-sex groups, 10 flies per food vial, for 5 days until reproductive maturity. At maturity, each sire was housed with three dams for 24 h. The following day, sires were discarded and each female was transferred to an individual fresh food vial for 5 days to allow oviposition, after which they were also discarded. We set up 20 sires per line, for a total of 60 sires and 180 dams.

Upon emergence, up to 10 randomly selected sons per dam were collected and housed together in single-sex vials for 5 days until reproductive maturity. After that period, four sons were mated with random virgin females from the same line. For logistic reasons, we spread out the number of paternal half-sibling families implemented across seven months representing flies collected from six different generations. For each generation, three half-sibling families per line were set up. Owing to these logistic constraints, we entered generation as a fixed effect in the model testing; this effect was not significant.

### (b) Trait measurement

To measure sperm number and length, four sons were each mated to a different virgin female and the sperm within the females subsequently dissected for analysis. We measured the head and flagellum length of six sperm of each type from each son, as previously described [25], with sperm collected from mated females ether-anaesthetized 2–4 h after mating.

### (c) Analysis

We analysed our data with ASREML. We obtained the different variance components in the full model and nested submodels for each trait separately and together (i.e. univariate and bivariate analysis, respectively) to determine significance of variance components. Nested submodels were obtained by constraining variances (univariate) or covariances (bivariate) of the full model to zero, which gives a new likelihood value. The statistical significance was assessed through likelihood ratio tests between the models. The asymptotic null distribution of this test is a chi-square with 1 degree of freedom (i.e. the number of parameters constrained to zero in the nested submodel).

We also calculated the coefficient of additive genetic variation (CV_A_), the coefficient of residual variation (CV_R_) and the evolvabilities, *e_*μ*_* (I_A_ in earlier literature), following Houle [11,12]. These provide a quantitative assessment of evolutionary potential. Note that while evolvability measures the expected percentage change in a trait for a unit strength of directional selection, it is not a measure of selection.

## 3. Results and discussion

All sperm length components had high narrow-sense heritabilities (table 1). Phenotypic variation and coefficients of additive genetic variation were high for short and long flagellum length, but low for short and long head length. Coefficients of residual variation were low for all traits. Evolvabilities are 10 times greater for short sperm traits than for long sperm traits, indicating that, in the absence of any constraints, parapserm have a much greater capacity for directional evolution than do fertilizing sperm. Within each sperm type, head and flagellum lengths were both positively phenotypically and genetically correlated (table 2), indicating both functional and genetic integration. Between types, most of the phenotypic correlations were significant (although low) and positive but none of the genetic correlations were statistically significant. Thus, the genetic architecture of *D. pseudoobscura* sperm indicates strong genetic integration within, but not between, sperm types.

**Table 1.** Basic descriptive univariate phenotypic and genetic data for the four sperm measurements. All measures made on 715 males distributed as four males per three dams per 60 sires. Data were slightly unbalanced at the level of sons. All heritabilities were significantly greater than zero.

	*X* (μm)	s.d.	*V*_A_	*V*_P_	*h*^2^ (s.e.)	CV_A_	CV_R_	*e_*μ*_*, (I_A, %_)
short sperm								
head	14.78	1.86	3.23	3.47	0.93 (0.25)	12.15	3.31	1.48
flagellum	76.86	9.87	67.21	97.71	0.69 (0.19)	10.67	7.18	1.14
long sperm								
head	60.36	2.53	4.34	6.43	0.67 (0.22)	3.45	2.40	0.12
flagellum	254.44	11.28	75.22	127.51	0.59 (0.22)	3.41	2.84	0.12

**Table 2.** Phenotypic correlations (above the diagonal) with significance provided parenthetically, and genetic correlations (below the diagonal) with SE provided parenthetically for head and flagellum length for both short sperm (SS) and long sperm (LS). Estimates statistically significantly greater than zero are in italics (*n* = 715).

	SS head	SS flagellum	LS head	LS flagellum
SS head	—	*0.75* (<0.001)	*0.22* (<0.001)	*0.09* (0.017)
SS flagellum	*0.85* (0.05)	—	0.04 (0.343)	0.07 (0.064)
LS head	0.26 (0.17)	−0.01 (0.21)	—	*0.29* (<0.001)
LS flagellum	0.14 (0.19)	−0.18 (0.21)	*0.61* (0.14)	—

The lack of strong genetic correlations between sperm types suggests a lack of developmental integration. Spermatogenesis is a shared process, but each sperm type is produced in separate sperm bundles. Together, these results suggest that each sperm type becomes specialized early in development. Specialization could occur either during the proliferation of germ cells or after the differentiation of germ cells. The former implies two populations of stem cells, one giving rise to short and one giving rise to long sperm. Alternatively, once germ cells differentiate into spermatocytes, transcription becomes active and different cell fates may be determined during this meiotic stage of spermatogenesis. This latter pattern is present in sperm heteromorphic Lepidoptera, where entry into the meiotic pathway signals divergence in the two sperm types [26]. However, in contrast to *Drosophila*, fertilizing and non-fertilizing sperm production in Lepidoptera is temporally shifted; spermatogenesis for fertilizing sperm starts in the larval period and stops after pupation, whereas non-fertilizing sperm are produced right before or after pupation and persists in the adult [26]. Future work will need to establish when and how the sperm type fates become differentiated in *D. pseudoobscura*.

The dominant hypothesis for the evolution of diverse sperm forms remains sperm competition resulting in sexual selection on sperm [3–7]. Yet, natural selection on sperm might be expected, given the need for efficient fertilization. Thus, as with other sexually selected traits, diversifying evolution may be limited by natural selection. Our results suggest that short sperm are more evolvable than long sperm but the patterns of historical selection of either sperm type is unknown. Whether short sperm historically have responded more quickly to selection is also unknown and needs to be tested across the *obscura* group, where all species so far examined are sperm heteromorphic [2]. Previous studies on both the *obscura* group [27,28] and other sperm heteromorphic taxa, including lepidopterans and stalk-eyed flies [29], found that the length of the short non-fertile morph was more phenotypically variable than the fertilizing morph. Indeed, some populations of *D. pseudoobscura* have been described as trimoprhic with two short classes of sperm (see [27,30]), although the function of the second short type has not been studied. The consistent pattern of low morphological variation in fertilizing sperm across different sperm heteromorphic taxa, each differing in sterile sperm development and phenotype, suggests the operation of either stabilizing selection for optimal fertilizing sperm size, and/or novel functions of sterile sperm that favour size variation. Neither of these hypotheses have been tested.

Determining the pattern of historical selection on sperm morphometry is difficult, because net selection can be influenced by a number of potential selective bouts, and the trait is an interacting phenotype subject to indirect genetic effects (i.e. sperm function in the female reproductive tract which is also under selection) [31]. A recent study of sperm monomorphic *D. melanogaster* compared inbred and outbred treatments of a population to determine past selection on sperm performance parameters [32]. The researchers found evidence for stabilizing selection on sperm length (see also [33]), despite experimental support for responses to directional selection [13]. The discrepancy between evidence for stabilizing selection, which should reduce genetic variation, and experimental response to directional selection demonstrating sufficient additive genetic variation, suggests sperm may have an intermediate length [32]. This intermediate length may arise as a consequence of trade-offs between sexual and natural selection that limit evolution. One interpretation of our results is that evolving heteromorphic sperm as independent modules may be one way out of this conflict, although this requires testing both within this species and across species.

We show here that sperm, despite sharing a common developmental process, can become developmentally and functionally non-integrated, evolving into separate modules that may facilitate independent responses to variation in the fertilization environment. By taking a quantitative genetics approach, this work has suggested testable hypotheses for both a mechanistic and an evolutionary understanding of sperm morphological evolution. Ultimately, understanding how evolutionary diversification in sperm is either facilitated or impeded requires additional quantitative genetic studies on sperm morphometry, including other such studies on sperm heteromorphic species, along with research aimed at determining past selection on sperm traits.

## Data accessibility

Data presented in the paper are deposited in the dryad database (doi:http://dx.doi.org/10.5061/dryad.c66v3).
